# Orientation of α-Synuclein at Negatively Charged Lipid Vesicles: Linear Dichroism Reveals Time-Dependent Changes in Helix Binding Mode

**DOI:** 10.1021/jacs.1c05344

**Published:** 2021-11-08

**Authors:** Sandra Rocha, Ranjeet Kumar, Bengt Nordén, Pernilla Wittung-Stafshede

**Affiliations:** ^†^Department of Biology and Biological Engineering and ^‡^Department of Chemistry and Chemical Engineering, Chalmers University of Technology, 412 96 Gothenburg, Sweden

## Abstract

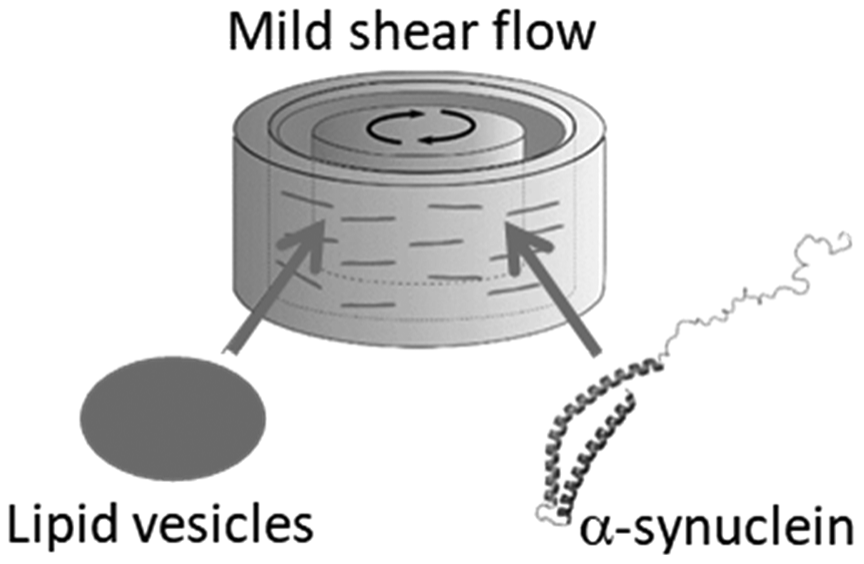

The neuronal protein α-synuclein, linked to Parkinson’s disease, binds to negatively charged vesicles adopting a partial α-helix structure, but helix arrangement at the vesicle surface is not fully understood. Using linear dichroism spectroscopy (LD), we study the interaction of monomeric α-synuclein with large unilamellar vesicles of 1,2-dioleoyl-*sn*-glycero-3-phospho-l-serine (DOPS), 1-palmitoyl-2-oleoyl-*sn*-glycero-3-phospho-l-serine (POPS), and 1,2-dioleoyl-*sn*-glycero-3-phospho-(1′-*rac*-glycerol) (DOPG) under mild shear flow. The LD data of oriented lipid vesicles show that the long axis of the protein helix is oriented preferentially perpendicular to the membrane normal but deviates from a uniform in-plane distribution. Upon initial binding, a fraction of helices are oriented in the direction of least curvature for all ellipsoid-shaped vesicles at a lipid:protein molar ratio of 100. However, at a lower protein concentration the helices distribute uniformly on DOPS and POPS vesicles. In all cases, the α-synuclein helices rearrange with time (minute time scale) in the shear flow and begin to tilt into the vesicle membrane. Faster reorientation kinetics in the presence of flow suggests that modulation of membrane dynamics, by thermal or shear-dynamic activation, may overcome steric barriers by what may be called “flow catalysis”.

## Introduction

Parkinson’s disease (PD), the most common neurodegenerative movement disorder, is neuropathologically characterized by the loss of dopaminergic neurons in the *substantia nigra* and by the presence of neuronal intracytoplasmic Lewy bodies, which are abnormal aggregates (amyloid fibrils) of the 140-residue protein α-synuclein.^1^ The function of α-synuclein is as yet not known, although the protein is suggested to regulate synaptic plasticity and neurotransmitter release.^2^ A role of α-synuclein in membrane/vesicle function is evidenced by the fact that the protein exists *in vivo* in both free cytosolic (unstructured) and membrane-bound (helical) states.^3−5^ Further characterization of membrane-bound α-synuclein will provide insights into the functional role of the protein, and in this context linear dichroism (LD) spectroscopy is a unique tool that may provide new information. LD is defined as the difference in the absorption between parallel and perpendicular linearly polarized light to a macroscopic orientation direction. Accordingly, LD spectroscopy provides information about the orientation of transition dipole moments of systems that are intrinsically oriented or are oriented during the experiment. Certain properties of a material may be detected only when the samples have some degree of orientation. LD has been applied to study transmembrane and surface-bound peptides or proteins by using shear-aligned vesicles.^6−10,32^ In a Couette flow cell, spherical vesicles, when subjected to shear flow, deform toward ellipsoidal shapes with preferred elongation along the flow lines.^11,12^ We recently showed that flow LD can be used to study α-synuclein bound to lipid vesicles.^13^

**Figure 1:**
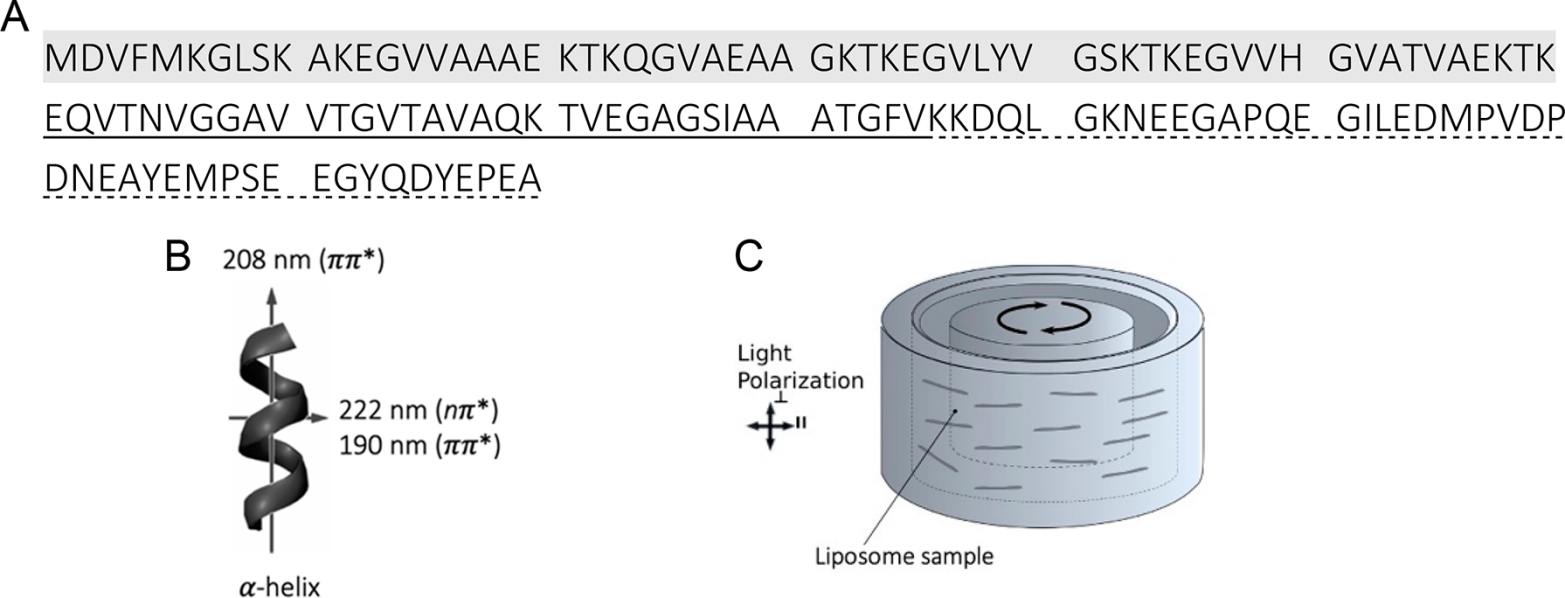
α-Synuclein sequence and principles of LD measurements. (A) α-Synuclein primary structure has three main regions: the N-terminal domain, highlighted in light gray, is rich in positively charged residues and determines the protein binding to negatively charged membranes; the nonamyloid-β component (NAC) domain, underline, forms the core of the amyloid fibrils, and the C-terminal domain, dotted underlined sequence, is rich in negatively charged residues. (B) Orientation of polarization transition moments in α-helix structure. (C) Couette flow cell, the alignment technique used in this study. Adapted with permission from ref (30). Copyright 2014 National Academy of Sciences.

Lipid–vesicle binding of synuclein triggers helix formation in the N-terminal part (residues 1–90).^14^ α-Synuclein has the highest affinity for negatively charged lipids, and in such systems the vesicle curvature does not seem to matter much.^15,16^ Binding of α-synuclein to negatively charged membranes is attributed to electrostatic interactions between lysine residues present in the N-terminal region of the protein (Figure 1) and acidic headgroups of lipids, but binding, albeit less efficient, is not completely abolished at high ionic strength conditions,^17−20^ indicating that hydrophobic interactions and entropy are also involved. Electron paramagnetic resonance (EPR) and angle-resolved second-harmonic scattering measurements support a model where the protein forms an extended α-helix structure at the membrane surface.^21−24^ However, data obtained from a solution NMR study of α-synuclein and small unilamellar vesicles (SUV) were inconsistent with this model, and instead multiple distinct binding modes that induce remodeling of the vesicles were proposed.^25^ While it is established that α-synuclein undergoes a structural change from unordered to α-helix when interacting with lipid vesicles,^17,18,26−29^ a consensus on the orientation and stability of the protein at the membrane has still to be reached.

Here, we used flow LD spectroscopy to probe the orientation of α-synuclein at the membrane surface of three different negatively charged vesicles (Figure 1). Our study points to a deviation of uniform in-plane distribution of α-synuclein upon initial binding to the on-average ellipsoid-shaped negatively charged vesicles, followed by time-dependent changes in the protein orientation (from flat on the surface to partial vertical membrane insertion).

## Experimental Procedure

### Protein

α-Synuclein was expressed and purified as described elsewhere.^13^

### Liposome Preparation

Negatively charged vesicles composed of 1,2-dioleoyl-*sn*-glycero-3-phospho-l-serine (DOPS), 1-palmitoyl-2-oleoyl-*sn*-glycero-3-phospho-l-serine (POPS), or 1,2-dioleoyl-*sn*-glycero-3-phospho-(1′-*rac*-glycerol) (DOPG) were prepared by the lipid film hydration method. Chloroform solutions of the lipids were purchased from Avanti Polar Lipids. Appropriate volumes of chloroform solution of the lipids were transferred to a round-bottom flask, and the organic solvent was evaporated by using a dry nitrogen stream. The resultant films were further dried under vacuum for at least 3 h and then hydrated with 10 mM phosphate buffer, pH 7.4, with 1 mM ethylenediaminetetraacetic acid (EDTA). The size of the liposomes was reduced by extrusion (Avestin LiposoFast-Basic extruder) using polycarbonate membranes of pore size 100 nm.

### Circular Dichroism Spectroscopy

Far-UV circular dichroism (CD) spectra of α-synuclein in the presence of increasing concentrations of liposomes were recorded on a Chirascan CD spectrometer (Applied Photophysics) from 260 to 190 nm by using a quartz cuvette with a path length of 1 mm, a bandwidth of 1 nm, and time per point of 0.8 s. Five individual spectra were acquired and averaged for each condition. Spectra of samples without the protein were subtracted from the CD signal of the α-synuclein. CD titration curves were fitted by using the one-step binding model F + Lipid_*L*_ ⇆ B(Lipid)_*L*_, where F is α-synuclein free in solution, B represents the protein fraction bound to the vesicles, and *L* is the number of lipid molecules interacting with one protein, as previously described.^26,27^

### Linear Dichroism (LD) Spectroscopy

The anisotropy of α-synuclein at fluid-phase bilayers was studied by using macroscopically oriented membranes. Linear dichroism (LD) spectra were recorded on a Chirascan CD spectrometer at a time per point of 0.7 s and a bandwidth of 1 nm by using a custom-made outer-cylinder-rotation Couette flow cell with an optical total path length of 1 mm (annular gap 0.5 mm) rotated at 1550 and 3100 s^–1^. Baselines at zero shear gradients were collected and subtracted from the spectra collected at different rotations. The LD measurements of α-synuclein membrane-bound state were done in a high-viscosity buffer (10 mM phosphate buffer containing 50% w/w sucrose) to reduce the light scattering of the liposomes by matching their refractive index.^6^ Retinoic acid was used as an internal membrane orientation probe,^31^ and the stock solution (5 mM) was prepared by dissolving the molecule in ethanol absolute. Retinoic acid was added to the liposome samples heated to 40 °C at a probe-to-lipid molar ratio of 1:200 (final volume of ethanol was <1% (v/v)), and the samples were equilibrated for at least 1 h prior to the measurements. The reproducibility of the results was confirmed by performing at least three independent experiments (with different batches of protein and lipid vesicles).

## Results

### Alignment of Negatively Charged Vesicles in the Fluid Phase

**Figure 2:**
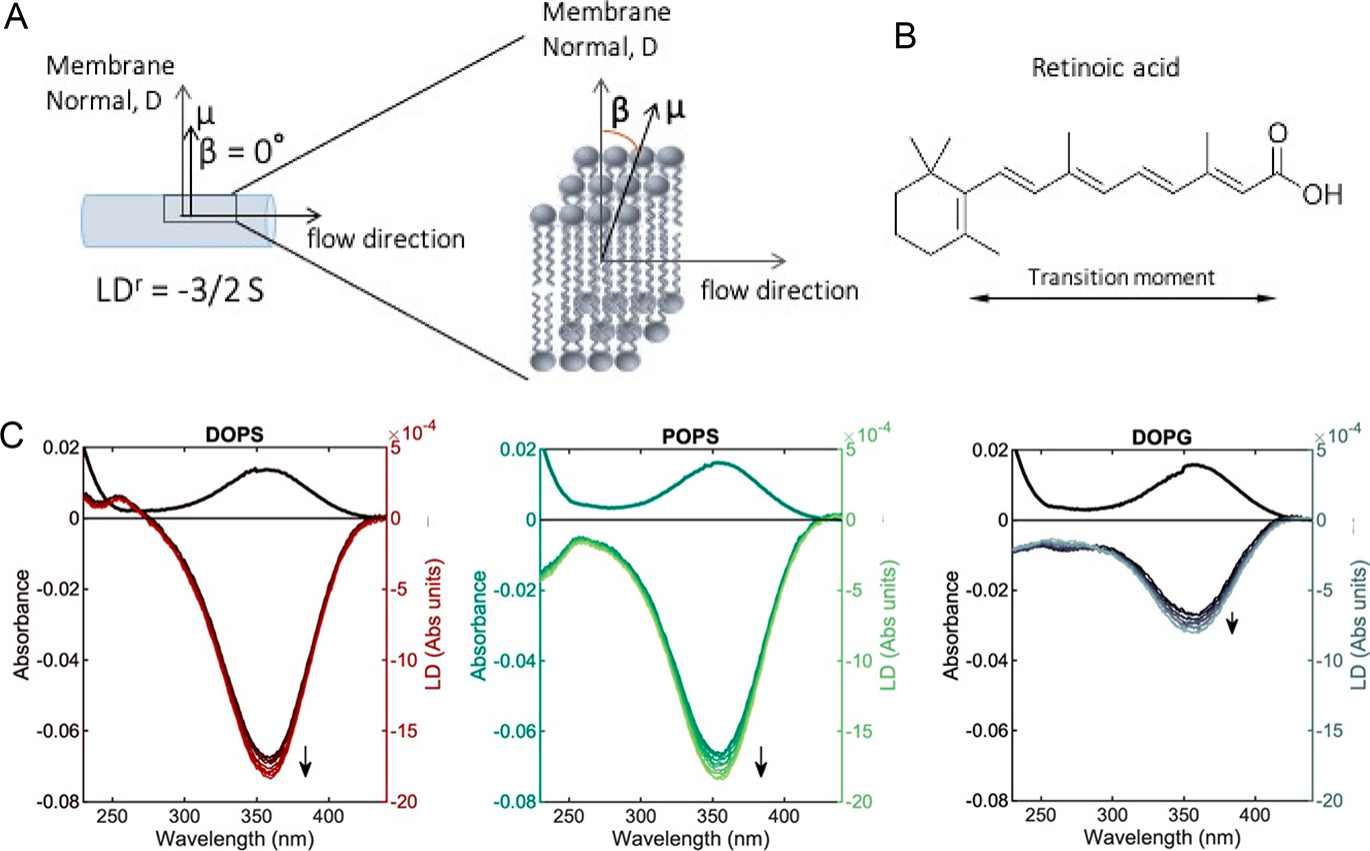
Flow alignment of negatively charged liposomes. (A) Right: molecular orientation relative to the membrane normal *D*. The overall orientation *S* of the deformed liposome pictured as a cylinder on the left is determined by using retinoic acid, which has β = 0° for the transition moment μ at 350 nm. Adapted with permission from ref (34). (B) Structure of retinoic acid membrane probe. The transition moment is oriented along the long axis of the molecule. (C) LD and absorbance spectra of retinoic acid incorporated into DOPS, POPS, or DOPG membranes in 50 wt % sucrose solution. Ten LD spectra were obtained sequentially over a time span of 30 min (the arrow indicates time).

Three systems were chosen, DOPS, POPS, and DOPG vesicles, to analyze the effect of different headgroups and degrees of unsaturation of acyl chains on α-synuclein orientation at membranes. Large unilamellar vesicles (LUVs) in sucrose buffer solution (50 wt %) were macroscopically oriented by flow with a Couette cell (Figure 1C). Deformation of a spherical liposome in shear flow results in an ellipsoid with the major axis at a small-angle relative to the flow direction when viewed in radial light-beam direction.^12,32,33^ The membrane normal is the molecular orientation reference direction of the membranes and gets preferentially more perpendicular to the long axis of the deformed vesicle and the flow direction with increasing flow (Figure 2A).^34,35^ Assuming a uniaxial local orientation distribution of the molecules around the membrane normal (*D*), the following relation between LD, defined as absorbance with light polarized parallel minus absorbance polarized perpendicular to flow direction, and order parameter *S*, is used to gauge the degree of macroscopic orientation of the liposome membrane^36^
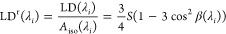
1where *A*_iso_ is the absorbance of the isotropic sample (in the absence of flow) and β(λ_*i*_) is the angle between the membrane normal *D* and transition moment of the chromophore; λ_*i*_ indicates the wavelength of light which determines what transition moment is being excited.^12,34,35,37^ The order parameter *S* describes the effective macroscopic orientation of the membrane normal *D* relative to a laboratory axis defined by the flow direction in the Couette cell^12,34,35,37^ and is 1 for perfect orientation and 0 for isotropic orientation. *S* = 1 would correspond to infinitely elongated liposomes aligned perfectly parallel with the flow so that *D* be perfectly perpendicular to the flow direction. The *S* factor of vesicles under flow at 3100 s^–1^ was determined by using retinoic acid as a probe (Figure 2B), which has been assigned β = 0° for the transition moment at 350 nm.^27^ The absorption maximum of retinoic acid is centered at 360 nm when incorporated into DOPS and DOPG membranes and at 357 nm in POPS bilayers (Figure 2C). The *S* factor was calculated from the LD and absorbance spectra by using eq 1: the value slightly increases from *S* = 0.081 to 0.088 over 30 min for DOPS, from 0.069 to 0.076 for POPS, and from 0.019 to 0.029 for DOPG liposomes when the samples are oriented under a shear gradient of 3100 s^–1^ in a Couette cell (Figure 2).

The degree of alignment (*S* factor) does not change much over time at the conditions studied here, indicating that the degree of vesicle deformation and orientation can be described as stationary. Deformation of liposomes subjected to Couette flow is generally extremely small, as indicated by an orientation far from perfect (*S* ≪ 1),^31,38^ and this is also seen here.

### Binding of α-Synuclein to Negatively Charged Vesicles

**Figure 3:**
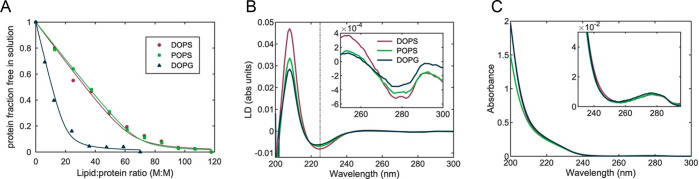
Binding and orientation of α-synuclein at negatively charged vesicles. (A) Fraction of α-synuclein free in solution as a function of L:P molar ratio upon titration of 4 μM of protein with DOPS (○), POPS (□), and DOPG (△) liposomes in phosphate buffer (pH 7.4). The fraction of free protein was calculated from circular dichroism data at 222 nm fitted with a single-step binding model (solid lines). (B, C) LD and absorbance spectra of α-synuclein (10 μM) immediately after addition to DOPS, POPS, and DOPG liposomes (1 mM) in 50 wt % sucrose solution. The inset graphs in (B) and (C) show magnified views of the tyrosine band at around 280 nm, and the dashed line in (B) indicates λ = 225 nm.

α-Synuclein forms an α-helix structure at pH 7.4 upon binding to DOPS, POPS, and DOPG liposomes in a concentration-dependent manner, and the circular dichroism at 222 nm is well described by a one-step binding model (Figure 3A).^26,27^ The stoichiometry of lipid–protein interaction, *L* (number of lipid molecules per one protein molecule), is 64 for DOPS, 68 for POPS, and 20 for DOPG liposomes, although the dissociation constant *K*_D_ is similar for the three systems (0.08 ± 0.03) × 10^–6^ M, in agreement with previous results.^27^ LD spectra of α-synuclein helices at the vesicle surface was collected at lipid:protein (L:P) molar ratios of 100 and 200, which are higher than *L* assuring that most protein is bound to the liposomes.

### Orientation of α-Synuclein Helices on the Surface of Vesicles

Assuming α-helices uniformly distributed in the plane of the surface of a deformed vesicle (ellipsoid), LD^r^ is described by modifying eq 1 to account for the arrangement and rotation average of transition moments in the helix:^36^
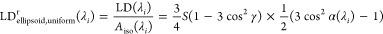
2where γ is here the angle between the membrane normal *D* and the long axis of the helix and α(λ_*i*_) is the angle between the long axis of the helix and the transition moment of the chromophore (selected at wavelength λ_*i*_) (Figure 1B). The n−π* transition moment (225 nm), which is perpendicularly polarized to the helix axis (α = 90°), contributes to both positive and negative LD due to helix rotation around its own axis. The observed negative LD at 225 nm is in qualitative agreement with α and γ both being close to 90°. However, the weakness of this transition, being prone to borrow intensity from neighbor transitions, makes it more straightforward to use the strong π–π* transition at 208 nm (polarized parallel to the long axis of the helix; i.e., α = 0) to determine the angles that α-synuclein helices make with the membrane normal. In the case the long axis of the helix is perfectly perpendicular to the membrane normal (γ = 90°), LD^r^/*S* at 208 nm will be +0.75.

**Figure 4:**
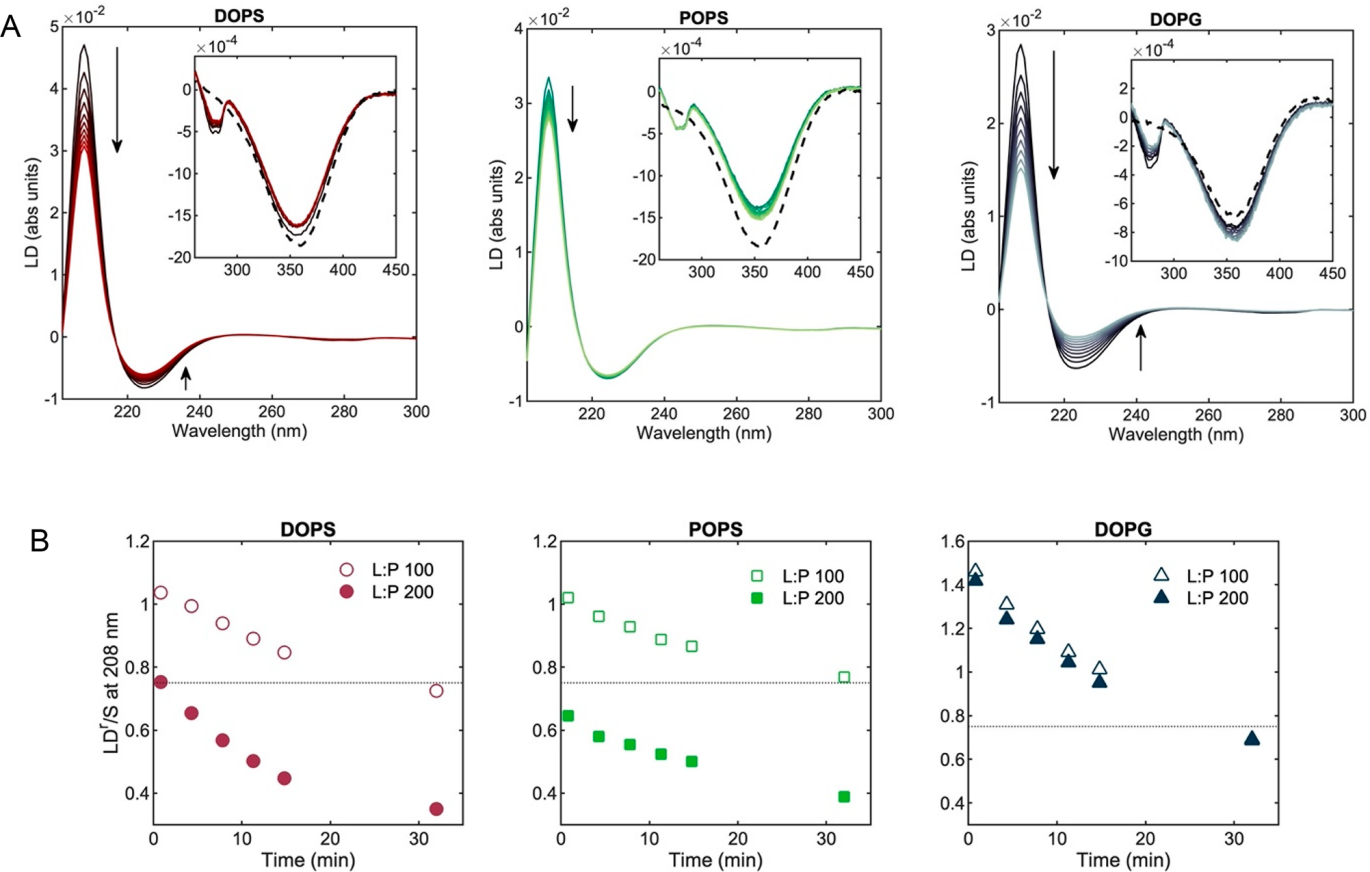
Time-dependent measurement of α-synuclein orientation at the membrane of negatively charged vesicles. (A) LD of α-synuclein (10 μM) in the presence of negatively charged vesicles (in 50 wt % sucrose solution) at a L:P molar ratio of 100 measured over 30 min under Couette flow. The arrows indicate time, and inset graphs show the retinoic acid LD band before (dashed black lines) and after addition of α-synuclein (solid lines). The LD of the tyrosine L_b_ band (276 nm) is visible after addition of the protein. (B) LD^r^/*S* at 208 nm of α-synuclein bound to vesicles at L:P molar ratios of 100 and 200 measured under flow over time (the points for L:P 100 and L:P 200 at 30 min overlap for DOPG). The dotted line indicates LD^r^(208 nm) = +0.75 in the case of uniform orientation of the long axis of the helix at an angle of 90° to the membrane normal.

α-Synuclein on membranes shows a positive LD band at 208 nm, which qualitatively indicates that the long axis of the α-helix structure is oriented more perpendicular than parallel to the membrane normal (Figure 3B). The *S* factor of the vesicles bound to the protein was obtained from the LD and absorbance spectra (Figure 3B,C and Figure S1) of retinoic acid (Table 1). The order parameter varies only little under flow as evidenced by the stable retinoic acid LD band, whereas the protein characteristic bands decrease in magnitude over 30 min (Figure 4A). Knowing the *S* factor (from retinoic acid), we calculated the LD^r^/*S* values at 208 nm, and they are significantly higher than +0.75 for all vesicles at L:P ratios of 100 and for DOPG at L:P 200 immediately after addition of α-synuclein but decrease when the samples are kept under flow (Figure 4B). Values higher than +0.75 immediately imply a deviation from the LD^r^ ellipsoid model (eq 2), and the only way to justify such high values is to assume a deviation from uniaxial distribution around *D* by some additional ordering of helices lying flat on the membrane surface preferentially along the long axis of the ellipsoidal vesicle (further discussed in the next section). Conversely, any inclusion of membrane insertion that results in helix tilting with respect to the surface plane would decrease LD^r^/*S*. Note that incomplete protein binding cannot explain the high LD^r^/*S* values: such a scenario would instead reduce the calculated LD^r^ values as, if so, *A*_iso_(λ_*i*_) in the denominator includes a contribution from the unbound protein. However, at the selected L:P ratios of 100 and 200, the lipid concentration is above saturation for all systems (Figure 3A), and thus all protein molecules are expected to be bound to vesicles. The measurements showed satisfactorily reproducible results, and the error was between 2% and 6%. Even if we assume extreme experimental errors in LD^r^ and *S* values, such as 10% in each, the LD^r^/*S* values could maximally go down from 1.46 to 1.17 (DOPG) and from 1.04 to 0.84 (for DOPG), which are still numbers significantly above 0.75.

**Figure 5:**
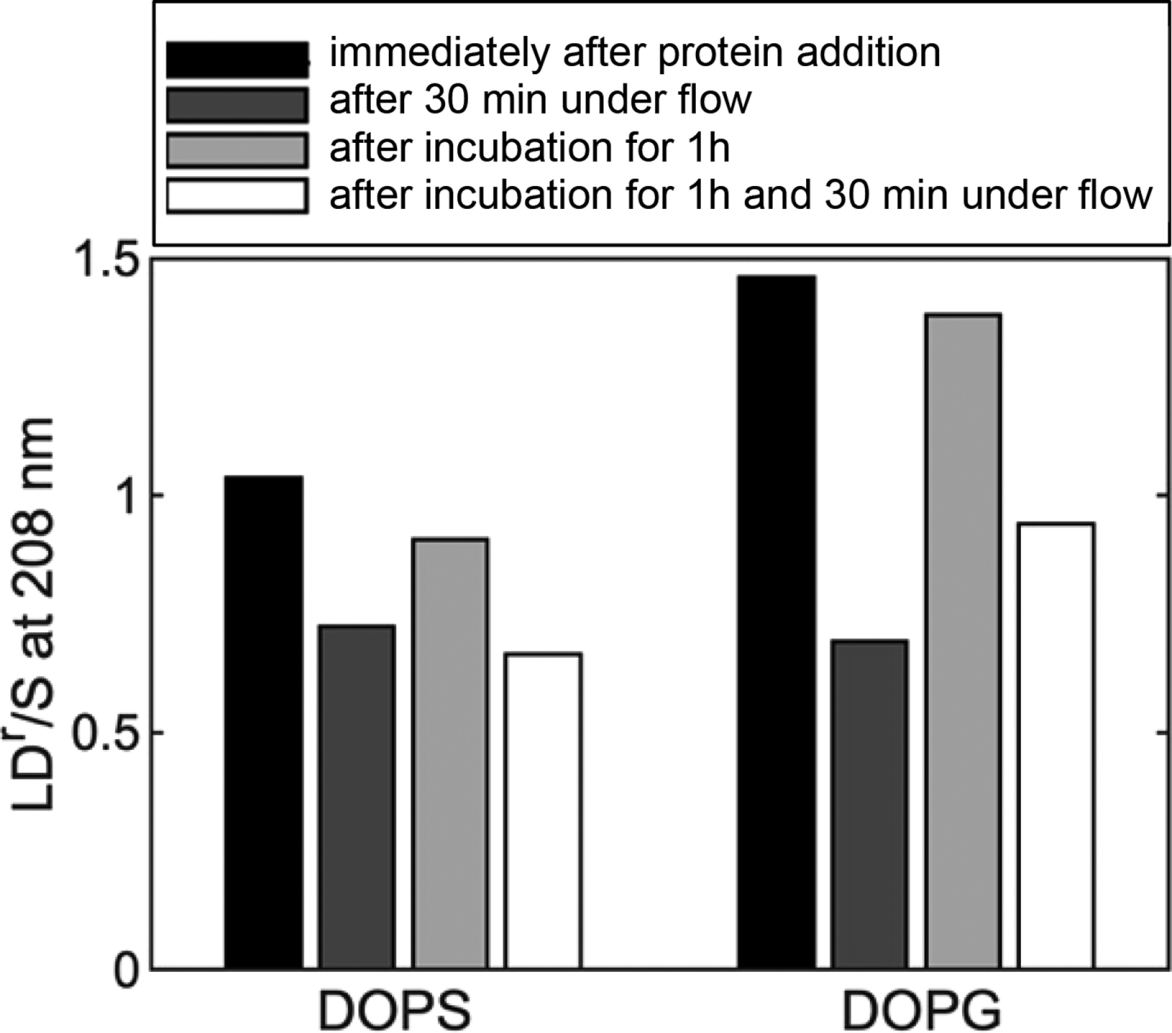
LD^r^/*S* at 208 nm of α-synuclein in the presence of DOPS and DOPG at a L:P ratio of 100. LD was measured immediately after addition of the protein to the vesicles and then after the sample was kept under flow for 30 min. In parallel, a sample was kept at rest after adding the protein for 1 h, and then LD was taken. This sample was then subjected to 30 min under flow, and then LD was taken again.

We also measured LD of vesicles incubated at quiescent conditions for 1 h after adding α-synuclein to DOPS and DOPG at a L:P ratio of 100. For such samples, when analyzed under flow, we obtained LD^r^/*S* (208 nm) slightly lower than the values measured immediately after protein addition but higher than the values found for samples kept under flow for 30 min (Figure 5). Adding 30 min of flow to such quiescent incubated samples resulted in a decrease in LD^r^/*S* similar to what was found for the samples immediately subjected to 30 min flow. Thus, the flow condition makes the LD^r^/*S* values decrease faster (Figures 4B and 5).

### From LD Data to Membrane Orientation of α-Synuclein

**Figure 6:**
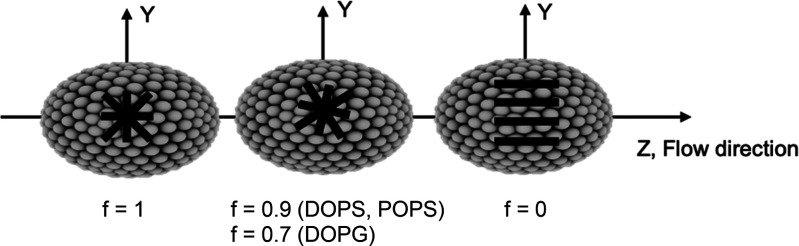
Schematic representation of α-synuclein distribution at the vesicle surface. On the left, the α-helices (represented by —) are uniformly distributed on the membrane plane. On the right, the other extreme case is shown where the protein molecules are aligned still flat on the surface but more parallel with the elongation direction (tube orientation). The proposed model to explain the high LD^r^/*S* values includes a fraction of the protein helices deviating from a flat uniform in-plane distribution and instead exhibiting in-plane bias of helix orientation parallel with the long-axis of the ellipsoid (drawing in the middle with derived fractions for the different lipid systems). *f* = fraction of helices with uniform in-plane distribution; 1 – *f* = fraction with biased orientation parallel with the flow (least curved direction on the ellipsoid).

Equation 2 can be used to fit only LD^r^/*S* values at 208 nm below +0.75, which corresponds to uniform helix arrangements parallel to the vesicle surface. All other arrangements of helix angles in relation to the vesicle surface will result in values lower than +0.75. Values higher than +0.75 are inconsistent with all possible uniform surface distributions but can be explained if some additional alignment bias is introduced. Therefore, we considered a model in which α-synuclein binds with the helix parallel to the vesicle surface with a distribution that is *not* uniform. Instead, we include that the helix on the surface favors orientations parallel with the deformation direction of the liposomes, i.e., the flow direction. The ellipsoid shape of the vesicles, induced by the flow, corresponds to more curvature perpendicular to the elongation direction and less curvature in the flow direction. Because of this nonspherical vesicle shape, an alignment bias of α-synuclein helices may be introduced. One way to explain the high LD^r^/*S* values is to assume higher probability for the helix to orient along the vesicle in the elongated, less curved direction. Such an orientation may be expected if the stiffness of the α-helix makes it sensitive to the curvature of the membrane such that it prefers a flatter arrangement. The limiting case for a biased alignment behavior is when α-helices are lying flat on the surface of an infinite tube and align themselves parallel with the tube (Figure S2). In this extreme tube case, LD^r^ should simply be^36^
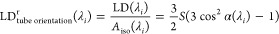
3where α is similar to α in eq 2 but here refers to the angle between the long axis of the fictive tube and the transition moment of the helix (the 208 nm transition will have α = 0 for parallel helix-tube alignment). LD^r^/*S* is +3 for a helix perfectly parallel to the tube. Having only the extreme tube case at our conditions is unrealistic since lipid vesicles deform poorly under flow (which is clear from our small *S* values). However, to account for the observed LD^r^/*S* values higher than +0.75, we may assume that a small fraction of protein helices on the ellipsoidal vesicles may exhibit tube-like orientation, while the rest of the helices are uniformly distributed on the ellipsoidal vesicles. To test this hypothesis, we created a two-state model as a hybrid between the ellipsoid and tube-alignment cases (with γ(208 nm) = 90°) defined by
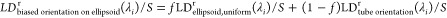
4where *f* is a measure of the width of the distribution, *f* = 1 corresponding to uniform distribution at the surface of the ellipsoid and *f* = 0 meaning perfect orientation parallel to tube. Ellipsoid and tube helix alignments are assigned the same macroscopic orientation factors *S*. In addition, all helices are assumed to lie flat on the membrane surface. LD^r^/*S* at 208 nm immediately after addition of the protein fitted with eq 4 gave *f* values of 0.9 for DOPS and POPS at L:P ratios of 100 and 0.7 for DOPG (both ratios). This indicates that the LD data can be explained by including a small fraction (1 – *f*) of α-synuclein helices being aligned parallel with the elongation direction (shown schematically in Figure 6) while the rest are uniformly distributed on the vesicle ellipsoid.

The above hybrid model was used to explain the unexpected LD^r^/*S* values of above +0.75. However, for LD^r^/*S* values below 0.75, the ellipsoid model (eq 2) can fit the data, and angles can be determined. Thus, eq 2 was used to analyze the data for all samples kept under flow for 30 min and for DOPS and POPS immediately after addition of α-synuclein at L:P ratios of 200. An LD^r^/*S* of 0.75 means a helix parallel to the vesicle surface, whereas values lower than +0.75 mean that the helix deviates from parallel alignment (i.e., it tilts with respect to the membrane surface). As judged from the angles derived from eq 2, the helix seems to (partially) tilt into the membrane over time, at least at some conditions. At 30 min under flow, the angle of the 208 nm transition with the membrane normal is 80° for DOPG vesicles at both L:P of 100 and 200. The angle is 84° for DOPS and 90° for POPS at L:P of 100 and 65° for DOPS and 66° for POPS at L:P of 200 after 30 min under flow (Table 1). Immediately after adding the protein, at a L:P of 200, the angle is 90° for DOPS and 78° for POPS. When considering possible experimental errors in the angles, only the data at L:P of 200 after 30 min under flow for DOPS (65°) and POPS (66°) show confident deviations from flat surface orientations of the helices. Apparent angles around 80° may simply reflect averaging effects of dynamics of peptide orientation or fluctuations of membrane surface. Taken together, the general trend for α-synuclein bound to these deformed vesicles (ellipsoids) is a time-dependent deviation from flat helix orientation on the membrane surface (with partial bias for the flow direction, most pronounced for DOPG vesicles) toward an arrangement that (at least in some cases) involves some bending/tilting of the helix in a direction perpendicular to the membrane plane.

**Table 1:** Order Parameter *S* of DOPS, POPS, and DOPG Vesicles before and Immediately after Addition of α-Synuclein (*t*_0_ Corresponds to the First Measurement, When the Flow Starts) and Angles of the Protein π–π* Transition at 208 nm (Helix Long Axis) Relative to the Membrane Normal for the Conditions That Could Be Fitted by Eq 2 (Ellipsoid Model); Angles Obtained Immediately after Adding the Protein (*t*_0_) and after 30 min under Flow (*t*_30 min_) Are Shown

	DOPS		POPS		DOPG	
*S* factor	L:P 100	L:P 200	L:P 100	L:P 200	L:P 100	L:P 200
vesicles only, *t*_0_	0.081	0.074	0.069	0.074	0.019	0.014
α-synuclein addition, *t*_0_	0.072	0.069	0.055	0.063	0.030	0.026
angle γ (deg) at 208 nm						
α-synuclein addition, *t*_0_		90		78		
α-synuclein addition, *t*_30 min_	84	65	90°	66	80	80

## Discussion

We here show that flow LD is a useful biophysical tool to investigate α-synuclein interaction with lipid vesicles. Considering the relatively mild flow applied, the vesicles do not disrupt but become deformed with a preferential elongation direction parallel with the flow. This makes possible the analysis of binding modes of molecules interacting with the vesicles, if the molecules have absorption from an assigned transition moment. Using this approach, we probed α-synuclein interactions with different negatively charged LUVs and made two discoveries.

First, we found that α-synuclein binds to negatively charged LUVs with an initial helix orientation that is biased toward an arrangement parallel with the long axis of the vesicle ellipsoid (i.e., parallel to the flow direction). This deviation from uniform distribution of helices is more pronounced for DOPG vesicles (LD^r^/*S* > +0.75 by many standard deviations even if large errors are assumed; based on three replicas, the error in LD^r^/*S* values is only 2–6%) and for high protein concentration for all vesicles (L:P ratio of 100 vs 200). We derived a simple hybrid model including biased behavior for a fraction of the protein helices (eq 4) that could successfully fit the high LD^r^/*S* values, and the results indicate that up to 30% (for DOPG vesicles; 10% for POPS and DOPS) of α-synuclein helices are in an orientation that is aligned parallel to the flow direction. High concentration of the protein at the vesicle surface may lead to the formation of small assemblies (multimers) at the membrane, and such helix–helix interactions may result in stiffer (and straighter) helices that in turn favor orientations in the direction of least curvature to make the most contacts with the lipid surface. In accord, α-synuclein monomers were shown to assemble into multimers with parallel helix interactions on small unilamellar vesicles.^39,40^ We note, however, that this concept is a speculation as we did not search for the presence of multimers in our experiments. Earlier binding studies of α-synuclein to negatively charged vesicles of different sizes (SUVs vs LUVs) did not reveal any dependence of affinity on the vesicle curvature.^15,16^ However, finer orientation preferences are not likely to be captured by affinity measurements. Further studies, including MD simulations of interactions with ellipsoids, would be desirable. The differences we observed in aS interaction behavior between PG and PS lipid vesicles may be related to the different L:P binding stoichiometries (64/68 for DOPS/POPS vs 20 for DOPG) and/or to differences in the properties of the membranes made of these two types of lipid vesicles (with PG vesicles being more fluid). Also here are further studies desired.

Second, we made the discovery that binding of α-synuclein to negatively charged vesicles is not a static event: over time in the mild shear flow, the helices rearrange from a flat-on-surface binding mode (with some directional bias) to partial insertion (tilting) into the membrane. The vesicles are intact and remain aligned throughout these experiments, and the helical content of α-synuclein does not change over time. We found α-synuclein orientation rearrangement to occur over a time scale of 30 min for all three lipid bilayers studied, though helix tilting or bending (i.e., deviation from flat surface orientation) was only confidentially determined for DOPS and POPS vesicles. From a structural point of view, it is not clear what the 24°–25° angular deviation from parallel-to-surface helix orientation means. The angle of the helix long axis with the membrane normal determined here (eq 2) assumes a single helix but may also represent an average where one part of the helix inserts itself more or less parallel to the membrane normal and the remaining part of the helix stays perpendicular. For example, our results for DOPS and POPS can be explained by 1/4 of the helix (i.e., around 20 residues) being vertically inserted into the membrane (and thus parallel to the membrane normal), whereas the remaining 3/4 of the helix stays flat on the membrane surface (vertical to membrane normal). Previous studies reported that α-synuclein forms an extended helix on LUVs,^21−24,41,42^ but wide-angle X-ray diffraction showed that the first 14 residues inserted vertically into POPG bilayers over time.^43^ Also, atomistic molecular dynamics simulations of α-synuclein in DOPS bilayers showed bending in the middle of the helix.^44^ Our results here for α-synuclein–membrane interactions, which depend on incubation time, protein concentration, and lipid chemistry, could unify multiple distinct binding modes described earlier.^25^ Incubation of samples without flow, followed by analysis in flow, reduced the α-synuclein helix rearrangement kinetics. Thus, by keeping vesicles in flow, resulting in deformation and modulation of membrane dynamics, it appears that kinetic and/or thermodynamic barriers toward α-synuclein reorientation are lowered. Although not an unexpected effect, such a “flow catalysis” has not been reported previously.

## Conclusions

Our LD data demonstrate preferential orientation of α-synuclein helices in the direction of vesicle least curvature and, with time, partial bilayer insertion of the helices. The exact protein distribution and orientation behavior on the membranes depend on lipid chemical structure, protein concentration, and time. The detected time-dependent changes and binding-mode variations may relate to α-synuclein’s physiological role in regulation of presynaptic vesicle activities. The detected interactions may also be related to pathology, as α-synuclein interactions with membranes (especially mitochondrial membranes) are thought to be involved in the initiation of Parkinson’s disease.^45^ Aberrant membrane interactions by α-synuclein may lead to rupture and leakage of membranes that may eventually cause cell death.^46,47^ Although our lipid vesicles here are not mimics of membranes or vesicles found *in vivo*, PG lipids play an important role in mitochondrial membranes whereas PS lipids are found in synaptic vesicle membranes.
