# Supplementary material for: Orientation of α-Synuclein at Negatively Charged Lipid Vesicles: Linear Dichroism Reveals Time-Dependent Changes in Helix Binding Mode
Source: J Am Chem Soc. 2021 Nov 8;143(45):18899–906. doi: 10.1021/jacs.1c05344 (PMC8603351; doi:10.1021/jacs.1c05344)
Supplement: Supplementary file 1 — ja1c05344_si_001.pdf [file ja1c05344_si_001.pdf]

# Orientation of α-synuclein at negatively charged lipid vesicles: linear dichroism reveals time-dependent changes in helix binding mode

Sandra Rocha[1]*, Ranjeet Kumar[1], Bengt Nordén[2], Pernilla Wittung-Stafshede[1]*
[1]Department of Biology and Biological Engineering, Chalmers University of Technology, Kemigården 4, Gothenburg, Sweden
[2]Department of Chemistry and Chemical Engineering, Chalmers University of Technology, Kemigården 4, Gothenburg, Sweden

*corresponding author: sandra.rocha@chalmers.se; pernilla.wittung@chalmers.se

## Supporting information

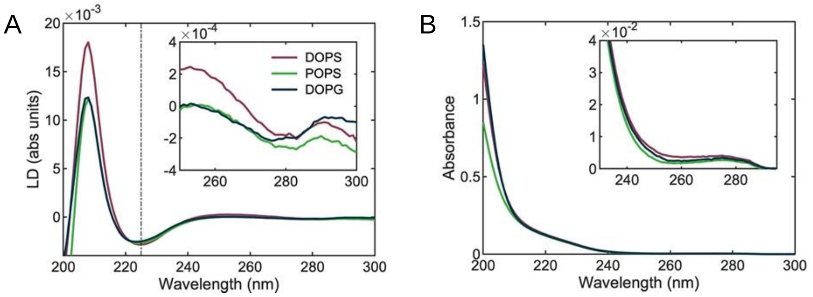


**Figure S1**: **Orientation of α-synuclein at the membrane of negatively charged vesicles at a lipid:protein molar ratio of 200.** LD (A) and absorbance (B) spectra of α-synuclein (5 μM) immediately after addition to liposomes (1 mM) in 50 wt % sucrose solution. The inset graph shows a magnified view of the tyrosine band at around 280 nm and the dashed line in A indicates $\lambda$ = 225 nm.

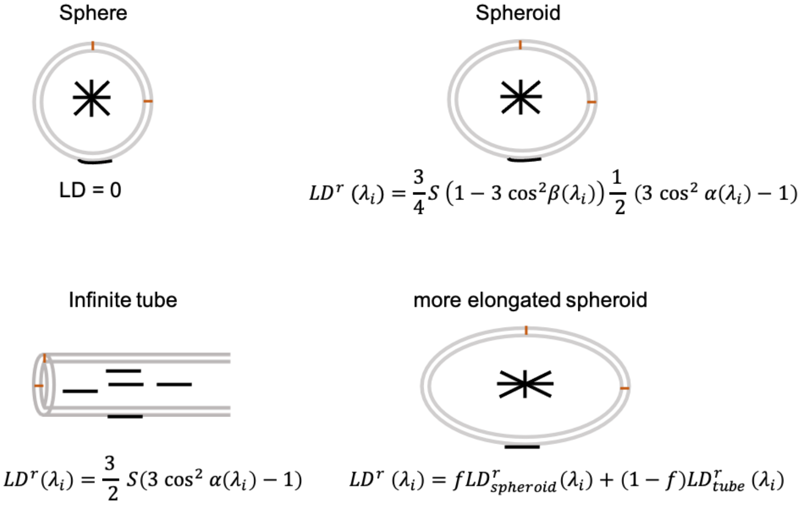


**Figure S2**: **Schematic representation of possible models of protein distribution at the surface of vesicles, spherical or deformed.** If the vesicles had remained perfectly spherical under the flow conditions, the LD signal would have been zero. The vesicles will deform under flow and for α-helices uniformly distributed at the surface of a spheroid, the arrangement and rotation average of transition moments in the helix need to be considered. An extreme case would be where α-helices are lying flat on the surface of an infinite tube and then align themselves parallel with the tube in order to optimize their contact area with the membrane. We propose that the protein orientation at the membrane of negatively charged vesicles may be described by a model that is hybrid between the spheroid and tube cases, thus spheroids that are so strongly deformed into a more elongated prolate configuration that α-helices are no longer uniformly distributed on the membrane plane.
